# Associations of bullying perpetration and peer victimization subtypes with preadolescent’s suicidality, non-suicidal self-injury, neurocognition, and brain development

**DOI:** 10.1186/s12916-023-02808-8

**Published:** 2023-04-12

**Authors:** Xue Wen, Yinuo Shu, Diyang Qu, Yinzhe Wang, Zaixu Cui, Xiaoqian Zhang, Runsen Chen

**Affiliations:** 1 Vanke School of Public Health, Tsinghua University, Beijing, 100084 China; 2 Institute for Healthy China, Tsinghua University, Beijing, China; 3 Chinese Institute for Brain Research, Beijing, 102206 China; 4 Department of Psychiatry, Tsinghua University Yuquan Hospital, Beijing, China

**Keywords:** Bullying, Subtype, Brain network, Suicide, NSSI

## Abstract

**Background:**

Although both peer victimization and bullying perpetration negatively impact preadolescents’ development, the underlying neurobiological mechanism of this adverse relationship remains unclear. Besides, the specific psycho-cognitive patterns of different bullying subtypes also need further exploration, warranting large-scale studies on both general bullying and specific bullying subtypes.

**Methods:**

We adopted a retrospective methodology by utilizing the data from the Adolescent Brain and Cognitive Development^SM^ Study (ABCD Study®) cohort collected between July 2018 and January 2021. Participants were preadolescents aged from 10 to 13 years. The main purpose of our study is to examine the associations of general and specific peer victimization/bullying perpetration with preadolescents’ (1) suicidality and non-suicidal self-injury; (2) executive function and memory, including attention inhibition, processing speed, emotion working memory, and episodic memory; (3) brain structure abnormalities; and (4) brain network disturbances. Age, sex, race/ethnicity, body mass index (BMI), socioeconomic status (SES), and data acquisition site were included as covariates.

**Results:**

A total of 5819 participants aged from 10 to 13 years were included in this study. Higher risks of suicide ideation, suicide attempt, and non-suicidal self-injury were found to be associated with both bullying perpetration/peer victimization and their subtypes (i.e., overt, relational, and reputational). Meanwhile, poor episodic memory was shown to be associated with general victimization. As for perpetration, across all four tasks, significant positive associations of relational perpetration with executive function and episodic memory consistently manifested, yet opposite patterns were shown in overt perpetration. Notably, distinct psycho-cognitive patterns were shown among different subtypes. Additionally, victimization was associated with structural brain abnormalities in the bilateral paracentral and posterior cingulate cortex. Furthermore, victimization was associated with brain network disturbances between default mode network and dorsal attention network, between default mode network and fronto-parietal network, and ventral attention network related connectivities, including default mode network, dorsal attention network, cingulo-opercular network, cingulo-parietal network, and sensorimotor hand network. Perpetration was also associated with brain network disturbances between the attention network and the sensorimotor hand network.

**Conclusions:**

Our findings offered new evidence for the literature landscape by emphasizing the associations of bullying experiences with preadolescents’ clinical characteristics and cognitive functions, while distinctive psycho-cognitive patterns were shown among different subtypes. Additionally, there is evidence that these associations are related to neurocognitive brain networks involved in attention control and episodic retrieval. Given our findings, future interventions targeting ameliorating the deleterious effect of bullying experiences on preadolescents should consider their subtypes and utilize an ecosystemic approach involving all responsible parties.

**Supplementary Information:**

The online version contains supplementary material available at 10.1186/s12916-023-02808-8.

## Background

Defined as the misuse of power during which one person (perpetrator) engages in repeated aggression against another (victim) that is intentional and involves an imbalance of power [1], peer bullying has adverse psychosocial impacts on participating parties’ mental health and [2] cognition [3, 4], with the long-term effect that can persist to later adulthood [5]. Determined by the intention embedded in the perpetrator’s actions, peer bullying can be overt, relational, or reputational. Overt perpetration is frequently manifested in the perpetrator’s actions to physically damage or threat of such damage to the victim. In contrast, relational perpetration aims to incur relational damage to the victim through harming peer relationships. Similarly, reputational perpetration also aims to inflict damage on the victim emotionally by damaging the victim’s reputation among peers [6–9]. To date, the prevalence of peer bullying in school-aged children was 30–60% [10], and the global prevalence was 9–32% [11–13]. It is alarming that the incidence rate of peer bullying has not been declining as expected globally in recent years, although many efforts have been made.

Specifically, ample research evidence has underscored both bullying victimization and peer perpetration as established risk factors for suicide ideation (SI), suicide attempt (SA), and non-suicidal self-injury (NSSI) [14–20], while these associations might be different across subtypes of peer bullying. For instance, previous studies have confirmed the predictive effect of direct victimization (i.e., overt) rather than indirect victimization (i.e., relational, reputational) on SI [9, 21–23], while inconsistent results exist [24, 25]. Meanwhile, according to a meta-analysis, relational victimization is strongly associated with higher risk of SI, while the association with SA is still controversial, and no study has yet found an association between relational victimization and NSSI [26]. Additionally, the predictive role of indirect aggression rather than direct aggression on NSSI among adolescents has been reported [27]. However, it is still worth noting that research on the relationship between bullying subtypes and NSSI and SA remains scarce.

Additionally, as one of the critical neurocognitive functions for building peer relationships [28], individuals’ executive function has been considered to be closely related to peer bullying [29–36, 20]. Herein, poor working memory was strongly associated with both bullying victimization and peer perpetration [37–39]. However, it remained unknown whether the same pattern exists in other domains of executive function (e.g., inhibition control, processing speed, emotion working memory) and other memory construct (e.g., episodic memory). Besides, such associations could differ among different subtypes of peer bullying [3]. Indeed, executive function deficits were uniquely associated with physical aggression, while better executive functions were associated with relational aggression [40]. Additionally, inhibition control was found to be significantly positively associated with relational aggression rather than physical aggression [38].

While there has been a strong interest in both the effects of peer victimization and bully perpetration, the related structural and functional brain abnormalities are not well understood. Relevant research is needed since chronic peer victimization during adolescence has already been proven to induce psychopathology-relevant deviations from normative structural brain development [41]. However, the potential association between bullying perpetration and brain structure remains unclear. Additionally, to the best of our knowledge, no research has yet explored their resting-state functional connectivity characteristics. Although a recent neuroimaging meta-analysis had underlined the change in neurobiological characteristics of victims, including altered brain structure and activated regions implicated in processing reward, social pain, emotion processing and regulation, social cognition, and risk-taking [42], there is still a need to observe whether such a change persists in a large sample.

In balance, there has been a dearth of research exploring the unique effects of different subtypes on peer bullying. Such a topic requires more research with large sample sizes, which is crucial for guiding interventions. Specifically, the associations between bullying subtypes and preadolescents’ self-harm behaviors (i.e., NSSI and SA), executive function, memory, and brain network characteristics need further exploration. Therefore, there are gaps in the existing literature that can be addressed, including elucidating the effects of general and specific peer bullying on preadolescents' suicidality/NSSI, neurocognition, brain structure, and brain function. To bridge these gaps, we aimed to (1) explore the influences of peer bullying and its subtypes on preadolescents’ SI, SA, and NSSI; (2) explore the influences of peer bullying and its subtypes on preadolescents’ executive function and memory; and (3) elaborate on the neuro-correlates of peer bullying, including brain structure and brain network. We hypothesized that different bullying subtypes might operate under distinct psycho-cognitive patterns in preadolescents. Additionally, given the accumulating evidence of the associations between bullying and abnormal psychological and cognitive functioning, we predicted the associations of bullying with disturbances in functional connectivity between or within neurocognitive brain networks.

## Methods

### Participants

We included data from the Adolescent Brain and Cognitive Development^SM^ Study (ABCD Study®) “Curated Annual Released 4.0 version.” Data for all included measures were collected at the 2-year follow-up assessment between July 2018 and January 2021, with the exception of demographic data being collected at baseline. For the origin sample conclude 10,414 participants, while preadolescents with incomplete information about our interested covariates were excluded, and participants with outlier BMI values (below 10 kg/m^2^ or above 50 kg/m^2^) were also excluded (see detailed information in Additional file 1: Fig. S1) [43]. Furthermore, only one individual from each family was selected at random to remove any possible effects of relatedness between subjects, resulting in a sample size of 5819 participants. Non-response analysis was conducted to explore the differences between those who were excluded from analyses and the final sample.

### Measurements

#### Exposures

The Peer Experiences Questionnaire (PEQ) was used to assess whether the child has either experienced overt, relational, or reputational victimization from peers or perpetrated overt, relational, or reputational aggression towards peers [44]. Each of these six domains was scored on a 5-point Likert scale ranging from 1 (“Never”) to 5 (“A few times a week”). The specific score of six bullying subtypes was calculated, and the general victimization score and general perpetration score were further obtained by summing up the domain scores. The present study only used data from the 2-year follow-up from the ABCD database since relevant data were only collected in that year.

#### Outcomes

##### Suicidality/NSSI

The child-report version of the suicide module from the computerized Kiddle Schedule for Affective Disorders and Schizophrenia (KSADS, Lifetime version) was used to assess children’s past and current SI, NSSI, and SA [45], while past and current diagnoses were collapsed into a single binary measurement.

##### Neurocognition

The emotional 2-back task was used to access preadolescents’ emotional working memory [46]; meanwhile, the NIH Toolbox Flanker task and Pattern Comparison Processing Speed task were used to access preadolescents’ attention inhibition and processing speed, with age-corrected standard scores used in final analyses [47]. All these measures have loaded onto a common component indicating the unity of executive function in preadolescents [48]. Besides, the NIH Toolbox Picture Sequence Memory task was further used to assess preadolescents’ episodic memory.

##### Brain structure

All preadolescents underwent structural magnetic resonance imaging (sMRI) according to standardized protocols, while the scanning parameters, pre-processing, and analytical pipelines are described elsewhere [46, 49]. The T1-weighted images acquired from 21 sites were processed at the Data Analysis, Informatics, and Resource Center (DAIRC) of the ABCD study. FreeSurfer v5.3 (http://surfer.nmr.mgh.harvard.edu) was used to process the locally acquired T1-weighted images and to estimate mean cortical thickness (CT), total cortical area (CA), and total cortical volume (CV). According to the Destrieux atlas, the cerebral cortex was parcellated into 74 regions in total [50]. Only scans that passed protocol compliance and quality control were used for corresponding analyses (see Table S1).

##### Brain network

Twenty minutes of resting-state functional magnetic resonance imaging (rsfMRI) were collected simultaneously. According to the Gordon parcellation accompanied by subcortical and cerebellar atlases, regions of interests (ROIs) were grouped into 12 predefined large-scale networks: (1) eight neurocognitive networks, including cingulo-opercular network (CON), cingulo-parietal network (CPN), default mode network (DMN), dorsal attention network (DAN), frontoparietal network (FPN), retrosplenial-temporal network (RTN), salience network (SN), and ventral attention network (VAN), and (2) 4 sensory networks, including auditory network (AUN), sensorimotor hand network (SMH), sensorimotor mouth network (SMM), and visual network (VN) [51]. Then, average time courses between each ROI were calculated, while average pairwise ROI correlations within and between each network were further computed. In total, 78 cortical (66 inter- and 12 intra-network correlations) network correlations were selected in our analyses, which were then calculated using the Fisher *r*-to-*z* transformation. Only scans that passed protocol compliance and quality control were used for corresponding analyses (see Additional file 1: Table S1).

##### Sociodemographic variables

Sociodemographic information, including children’s age, sex, race/ethnicity, marital status, parental education level, combined household income, and data acquisition site, were collected. The highest level of parental attained education was selected and recoded into five categories (i.e., Below High School, High School Graduate/GED, Some College, Bachelor Degree, and Postgraduate Degree). Moreover, household income was recoded into three levels (i.e., < 50 K, ≥ 50 K and < 100 K, and ≥ 100 K USD). All these adjustments have been implemented in previous studies [52, 53].

### Statistical analysis

All statistical analyses were performed using R (version 4.2.1). The associations of peer bullying (including both the victimization dimension and perpetration dimension) with suicidality/NSSI, neurocognition, and brain development in preadolescents were investigated. Since the dependent variables conclude both binary variables (i.e., SI, NSSI, and SA) and continuous variables (cognitive performances, brain morphometrics, and functional connectivities), generalized linear mixed models were performed using R package glmmTMB (logit link) and lmeTest (identity link) respectively, with data acquisition site modeled as a random intercept [54, 55]. All the continuous variables were standardized, including age, income, education, and BMI. Other categorical variables were dummy coded before being included in the model, including sex, race/ethnicity, marital status, and handedness. The conditional R-square for each model was calculated.

For sMRI analysis, only 4954 scans that passed the quality control were taken into the subsequent analyses. Given no hypotheses regarding lateralized effects, values for the right and left hemispheres were summed in our study. The associations between bullying, peer bullying, and global brain morphometrics were first explored, including mean CT, total CA, and total CV. Additionally, to determine which regions were associated with peer bullying, 74 (regions) *3 (brain morphometrics, including CT, CA, and CV) linear mixed models were conducted to examine all regions in each modality. To control for multiple testing across regions, false discovery rate (FDR) was further used through R package stats [56]. For brain network analyses, 3830 qualified scans were included. The associations between peer bullying and (1) within-network connectivity for 12 Gordon networks (12 FDR-corrected comparisons) and (2) between-network connectivity (11 FDR-corrected comparisons per network) were examined.

### Covariables

For behavior and neurocognition analysis, age, sex, race/ethnicity, BMI, and SES (i.e., marital status, income, and parental highest education) were considered as covariables. For sMRI analysis, handedness and intracranial volume (ICV) were further added as covariates, while handedness and mean motion were further added as covariables in brain network analysis. BMI and SES variables were added as additional covariates in accordance with prior research showing associations of those factors with brain structure and brain network in preadolescents [53, 57–61].

## Results

A summary of the sociodemographic characteristics of the analyzed sample can be found in Table 1 and Additional file 1: Table s2. In comparison to those excluded from analyses, the final sample was younger in age and had a lower proportion of females, a lower proportion of racial/ethnic minority status individuals, a higher proportion of household married, and higher parental education.

**Table 1:** Demographic characteristics of the analyzed sample and the excluded sample

**Characteristic**	Final sample (*N* = 5819)	Excluded (*N* = 4595)	*P* value^a^
Age, mean (SD), years	11.9 (0.6)	12.1 (0.7)	< 0·001
Sex			0.046
Male	3098 (53.2)	2355 (51.3)	
Female	2721 (46.8)	2240 (48.7)	
Race/ethnicity			< 0·001
Non-Hispanic White	3347 (57.5)	2255 (49.1)	
Non-Hispanic Black	643 (11.1)	779 (17.0)	
Hispanic	1122 (19.3)	964 (21.0)	
Asian	128 ( 2.2)	88 ( 1.9)	
Other	579 (10.0)	509 (11.1)	
Household married			< 0·001
Married	4141 (71.2)	3044 (66.2)	
Unmarried	1678 (28.8)	1485 (32.8)	
Household income			0.093
< 50 k	1590 (27.3)	1097 (29.1)	
≥ 50 k and < 100 k	1718 (29.5)	1051 (27.9)	
≥ 100 k	2511 (43.2)	1618 (43.0)	
Parental highest attained education level^b^			< 0·001
Below High School	211 ( 3.6)	252 ( 5.5)	
High School Grad/GED	423 ( 7.3)	487 (10.6)	
Some College	1412 (24.3)	1208 (26.4)	
Bachelor Degree	1596 (27.4)	1146 (25.0)	
Postgraduate Degree	2177 (37.4)	1490 (32.5)	
BMI, mean (SD)	20.59 (4.84)	20.56 (4.90)	0.811

^*a*^Differences among two groups were calculated with *t*-tests and *χ*^*2*^ tests

^*b*^GED, graduate equivalency degree

### Association between peer bullying and preadolescents’ suicidality/NSSI

**Fig. 1:**
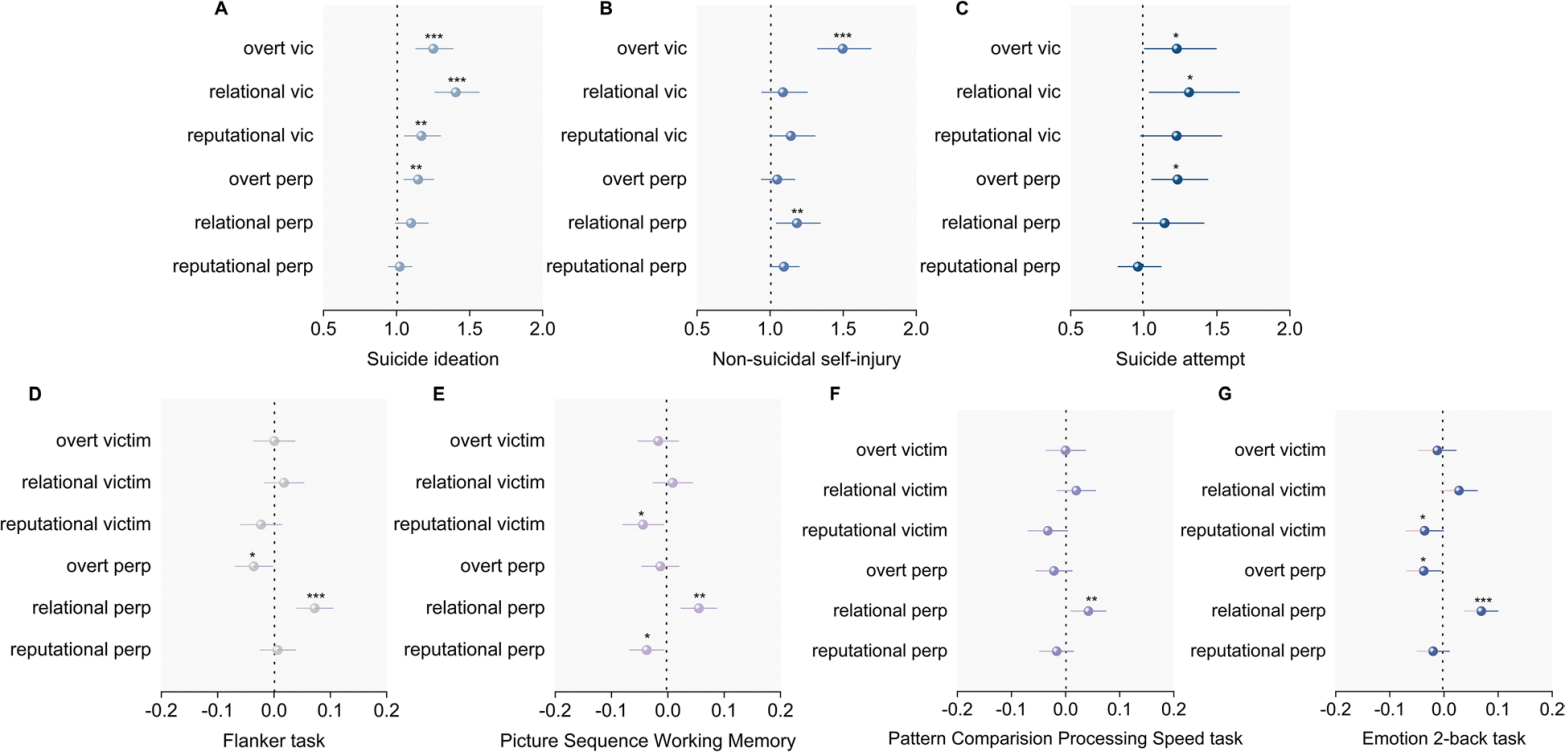
Associations of bullying subtypes with suicidality/NSSI and neurocognition in preadolescents. Adjusted for age, sex, race/ethnicity, site, BMI, marital status, income, and parental highest education. (1) **A**–**C** Odds radio was reported for binary suicidality/NSSI variables; (2) **D**–**G** coefficient was reported for continuous cognitive variables. vic, victimization; perp, perpetration

First of all, we sought to delineate the association of peer bullying with SI, NSSI, and SA. Both general victimization and general perpetration were found to be negatively associated with higher risks of SI, NSSI, and SA (Table 2). With regard to specific subtypes, all three types of victimization (i.e., overt, relational, reputational) were shown to be associated with higher risks of suicidality/NSSI. Besides, overt perpetration was positively associated with suicidality (including SI and SA), while relational and reputational perpetration performed significant positive associations with NSSI (Fig. 1 and Additional file 1: Table s3).

**Table 2:** Associations of peer bullying with suicidality/NSSI and cognition in preadolescents

**Predictor**		**95% CI**		
	**Estimate**	**Lower**	**Upper**	***P* value**
**Suicidality/NSSI**^**a**^				
Suicide ideation				
Victimization	1.79	1.64	1.95	< .001
Perpetration	1.22	1.12	1.33	< .001
Non-suicidal self-injury				
Victimization	1.66	1.50	1.83	< .001
Perpetration	1.30	1.18	1.43	< .001
Suicide attempt				
Victimization	1.74	1.50	2.03	< .001
Perpetration	1.26	1.09	1.45	0.001
**Neurocognition**^**b**^				
Flanker task				
Victimization	− 0.005	− 0.036	0.026	0.762
Perpetration	0.040	0.008	0.071	0.013
Picture Sequence Memory task				
Victimization	− 0.044	− 0.075	− 0.014	0.005
Perpetration	0.012	− 0.018	0.043	0.433
Pattern Comparison Processing Speed task				
Victimization	− 0.013	− 0.044	0.019	0.431
Perpetration	0.010	− 0.021	0.042	0.526
Emotion 2-back task				
Victimization	− 0.017	− 0.047	0.012	0.256
Perpetration	0.017	− 0.013	0.047	0.255

^a^Odds radio was reported for binary suicidality/NSSI variables

^b^*β* coefficient was reported for continuous neurocognition variables

Adjusted for age, sex, race/ethnicity, site, BMI, marital status, income, and parental highest education

### Association between peer bullying and preadolescents’ neurocognition

Next, the association between peer bullying and cognition was explored. Significant negative associations between general victimization and cognitive performances in the Picture Sequence Memory task were found (*β* =  − 0.044, *P* = 0.005). However, there is a significant positive association between general perpetration and Flanker task scores (*β* = 0.040, *P* = 0.013). Notably, across all four tasks, significant positive associations between relational perpetration and better executive function and episodic memory were consistently shown (*P* < 0.05), while opposite patterns were shown in overt perpetration (Fig. 1 and Additional file 1: Table s4).

### Association between peer bullying and preadolescents’ brain structure

**Fig. 2:**
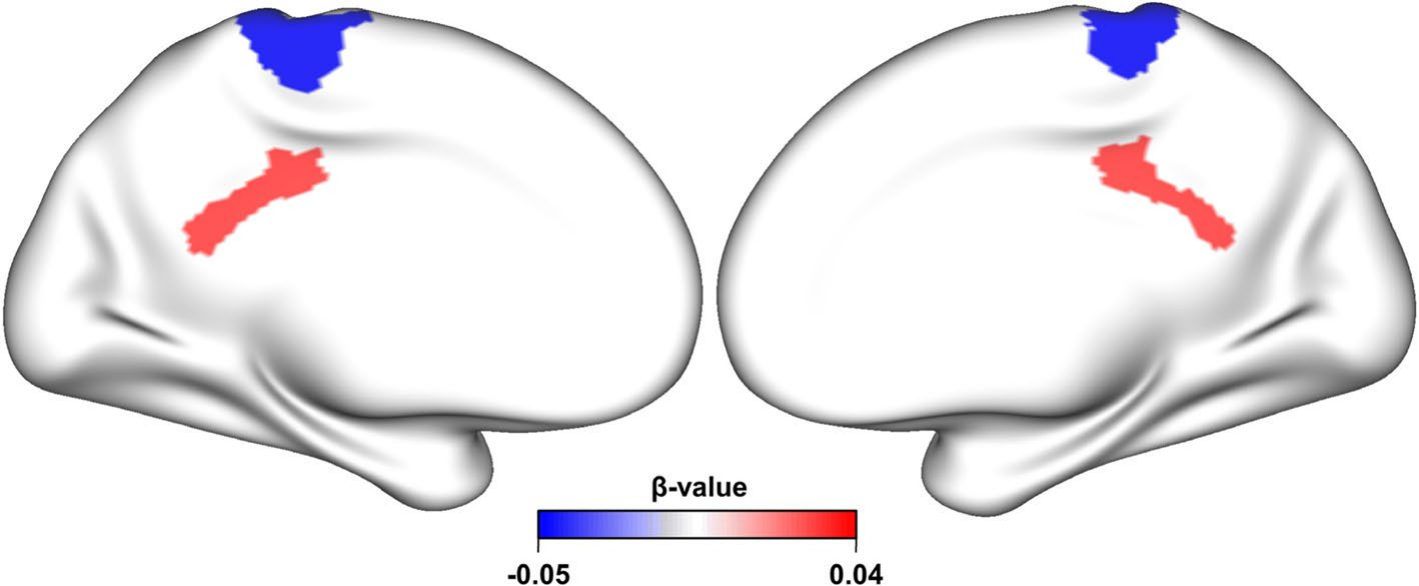
Association of brain structure with victimization. After adjusting for age, sex, race/ethnicity, site, handedness, BMI, SES, and ICV, victimization was found to be positively associated with total cortical area of bilateral posterior dorsal part of the cingulate gyrus, while negatively associated with mean cortical thickness of bilateral paracentral lobule and sulcus

We also elucidated the brain structures of peer bullying. We found a significant negative association between victimization and bilateral CT in the paracentral lobule and sulcus (*β* =  − 0.052, *P*_*fdr*_ = 0.047) and an approximately significant positive association between victimization and bilateral CA in the posterior dorsal part of the cingulate gyrus (*β* = 0.044, *P*_*fdr*_ = 0.045), as shown in Fig. 2 and Additional file 1: Table s5.

### Association between peer bullying and preadolescents’ brain network

**Fig. 3:**
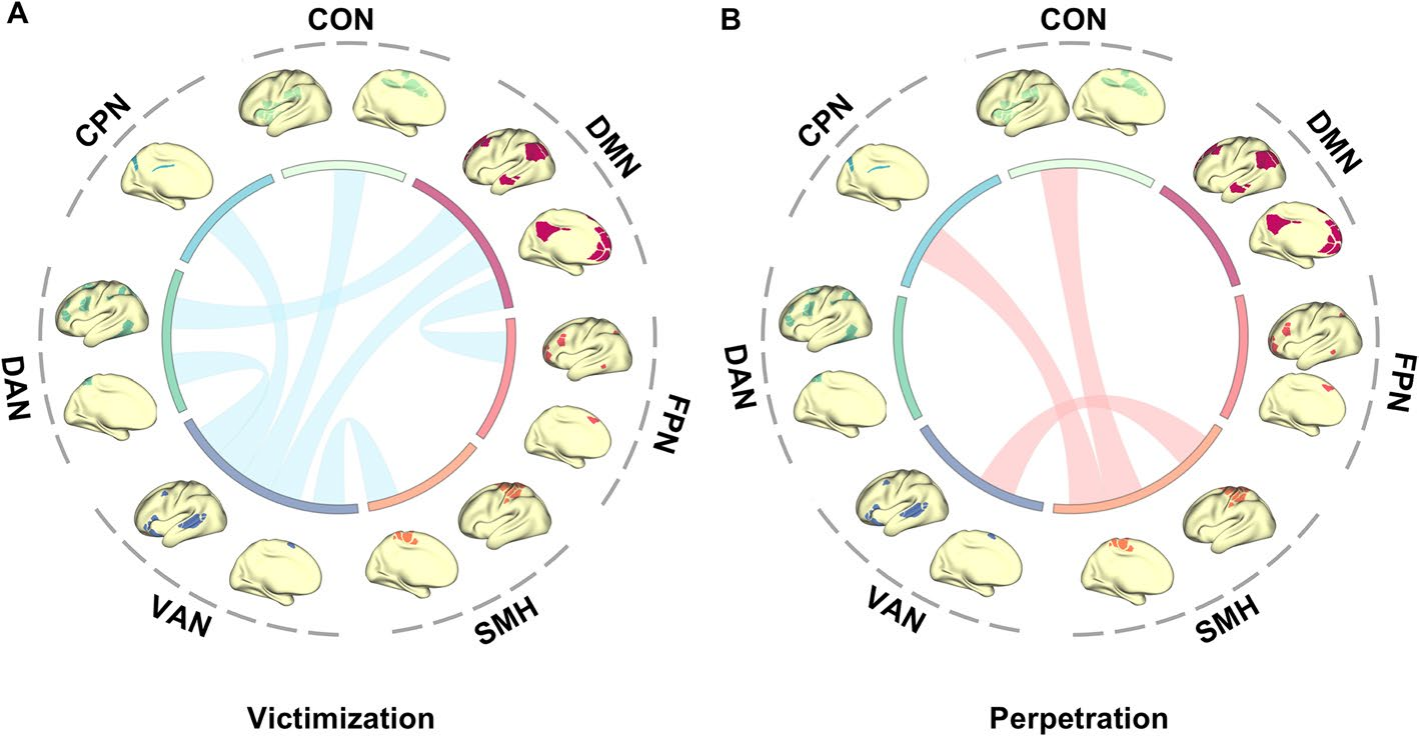
Associations of resting-state functional connectivity with victimization and perpetration. **A.** After adjusting for age, sex, race/ethnicity, site, handedness, and mean motion, significant associations between victimization and DMN-FPN, DMN-DAN, VAN-DMN, VAN-DAN, VAN-CPN, VAN-CON, and VAN-SMH were found. **B.** As for perpetration, significant associations were found in SMH-CON, SMH-VAN, SMH-CPN (DMN, default mode network; FPN, fronto-parietal network; SMH, sensorimotor hand network; VAN, ventral attention network; DAN, dorsal attention network; CPN, cingulo-parietal network; CON, cingulo-opercular network)

Specific network-level connectivity patterns were shown to be significantly associated with both victimization and perpetration. Notably, victimization seems to be associated with functional connectivity between DMN and DAN (*β* = 0.048, *P*_*fdr*_ = 0.022) and between DMN and FPN (*β* = 0.050, *P*_*fdr*_ = 0.024). Five functional connectivity related to VAN have also shown to be associated with victimization, including (1) DMN (*β* =  − 0.065, *P*_*fdr*_ = 0.004); (2) DAN (*β* = 0.041, *P*_*fdr*_ = 0.045); (3) CON (*β* = 0.051, *P*_*fdr*_ = 0.018); and (4) CPN (ventral: *β* =  − 0.057, *P*_*fdr*_ = 0.012). The functional connectivity between the VAN and SMH was associated with both victimization (*β* = 0.045, *P*_*fdr*_ = 0.044) and perpetration (*β* =  − 0.056, *P*_*fdr*_ = 0.015) while in the opposite direction. Besides, perpetration was also found to be associated with other SMH-related function connectivities, including CON (*β* = 0.053, *P*_*fdr*_ = 0.015) and CPN (*β* = 0.059, *P*_*fdr*_ = 0.015), as shown in Fig. 3 and Additional file 1: Table s6).

## Discussion

This is the first study investigating the effects of peer bullying and its subtypes on preadolescents’ suicidality/NSSI, executive function and memory, brain structure, and brain network with a large sample size. Our findings provided four clinically informative insights on (1) higher risk of SI, NSSI, and SA associated with bullying perpetration/peer victimization and their subtypes (i.e., overt, relational, and reputational) in preadolescents; (2) association between poor episodic memory and victimization, especially for reputation victimization; (3) association between better executive function and general perpetration, yet distinct pattern between overt perpetration and relational perpetration; (4) associations of victimization with structural brain abnormalities in the bilateral paracentral cortex and posterior cingulate cortex; (5) associations of victimization with brain network disturbances between DMN and DAN, between DMN and FPN, and VAN-related functional connectivities (i.e., DMN, DAN, CON, CPN, and SMH); and (6) associations of perpetration with brain network disturbances between VAN and SMH.

### Peer bullying and mental health development

Our study found the positive associations of all types of victimization on preadolescents’ suicidality, which is largely consistent with previous studies [26]. Notably, relational victimization was only associated with self-harm behaviors with suicide intention, suggesting the relationship between relational victimization and SA might be primarily driven by increased odds of SI. Relatively, only overt perpetration showed an association with suicidality. It may be the case that the victim’s responses might reinforce the perpetrator’s overt aggressive behaviors (e.g., retaliation, crying, or withdrawing) [62, 63], which could impose worse impacts on preadolescents. Besides, previous research has consistently shown that individuals’ self-harm and other-harm behaviors (i.e., aggressive behaviors) co-occur across various populations (known as “dual-harm”) [64, 65], and those who performed dual-harm exhibit significantly higher levels of psychopathology [66]. Additionally, indirect perpetration (i.e., relational, reputational) has been shown to be associated with NSSI in our study, which mirrored a previous study [27]. This might be explained by the transition of aggression strategy with age, as children develop in their social understanding and social ability. One possible piece of evidence could be the increased tendency for indirect aggression and decreased tendency for overt aggression in children when they grow up [67].

### Peer bullying and neurocognition development

The association between general victimization and lower executive function was not found in our study, which is inconsistent with a recent study also conducted using ABCD data [20]. This might due to the differences in the way bullying was measured, as parent-reported dichotomous victimization (yes or no) variable based on KSADS was used in Menken et al.’s study, whereas continuous victimization and perpetration variables measured by total score of 18 items in the self-reported PEQ questionnaire were used in our study, and we included both victimization and perpetration in the GLMM model in order to obtain a clearer picture of the relationship between peer bullying and children’s developmental outcomes. However, reputation victimization has shown to be associated with impaired working memory according to our findings. Our findings also confirmed the association between general victimization and preadolescents’ poor episodic memory, especially for reputational victimization. Rumination may play a crucial role in this association, as it disrupts adolescents’ episodic memory, [68] and adolescents with rumination tend to behave in a clingy, needy, yet hostile manner, which creates a breeding ground for reputational victimization [69].

Additionally, general perpetration was found to be associated with better executive function. Compared to the negative association between overt perpetration and neurocognition, relational perpetration has performed positive association with better episodic memory (Picture Sequence Memory task) and executive function, including inhibition (Flanker task), processing speed (Pattern Comparison Processing Speed task), and emotion working memory (Emotion 2-back task). Indeed, substantial heterogeneity of this association has been observed across studies due to methodological issues, such as small sample sizes, age differences in the target group, and the selection of covariates [3, 37–40]. To the best of our knowledge, this is the first large-population study to examine the associations of executive function and memory with all subtypes of peer bullying in preadolescents. As shown in the previous study, relational perpetration was considered as a more cognitively engaged and covert way of expressing aggression [70], while preadolescents with relational perpetration performed higher social information processing and social intelligence [71, 72]. Therefore, to achieve their goal wisely and successfully, relational perpetrators not only need to be flexible in using their social skills but also need to reasonably inhibit their immediate aggressive impulses, which might explain their better executive function and episodic memory. Otherwise, individuals with better neurocognition skills might also tend to choose relational perpetration rather than overt perpetration to reach a higher social hierarchy and gather more social capital among peers.

### Peer bullying and brain development

Our further work extended the psycho-cognitive characteristics of peer bullying by elucidating its brain signatures. Victimization has been shown to be associated with structural abnormalities of both the paracentral cortex and posterior cingulate cortex (PCC). At the same time, victimization was richly associated with functional connectivities between several neurocognitive networks (e.g., DMN, DAN, VAN), which might serve as a common biomarker for vulnerability to mental disorders [73–75]. Accumulating evidence reveals the association of suicide with the structural and functional abnormalities of the PCC, forming a key hub for cognitive control and serving as a crucial part of the DMN [76–78]. Indeed, as shown in previous research, DMN has been considered to play an important role in episodic retrieval, regulating internally directed cognition (e.g., future imaging events), and self-referential processing [79, 78, 80]. Additionally, FPN might aid the memory retrieve process by representing retrieval targets and prioritizing relevant environmental cues [79, 81]. Notably, interactivity between DMN and FPN was found to be associated with episodic retrieval [79, 82]. Current study suggests that victimization was strongly associated with functional connectivity between DMN and FPN, similar to a recent study suggesting the association of gamma connectivity between DMN and FPN with suicide risk [83]. Thus, the association between impaired episodic memory and victimization might be closely related to disturbed functional connectivity between DMN and FPN. Meanwhile, the DMN-DAN anticorrelation presented optimal allocation of the cognitive resource by the brain and thus was always considered a helpful property in the human brain [84]. However, decreased DMN-DAN anticorrelation was linked to multiple neurological and psychiatric disorders [85–89]. Combined with the decreased DMN-DAN anticorrelation discovered in our study, it might partially explain suicidality/NSSI and neurocognition deficits associated with victimization.

Notably, the current study highlights the associations of victimization with disrupted interaction related to the VAN and DMN, DAN, CON, CPN, and SMH. As shown in the previous study, impaired attention network function was associated with psychopathology in children since functional connectivities between VAN and DAN, CON were found to be significantly associated with children’s social, thinking, and attention difficulties [90]. Additionally, among children with a history of depression or anxiety, abnormal attention network functioning was found to be closely related to their “attention bias” (i.e., changes in attention to stimuli of negative valence) but not to their current psychopathological symptoms [91]. Meanwhile, such “attention bias” was also shown to be associated with children’s suicide risk or NSSI behavior [92–94]. Additionally, the CPN has been consistently shown to be associated with executive function [95]. Thus, the neurobiological basis underlying the associations between executive function deficits and victimization might be the disturbed functional connectivities between VAN and CPN.

The above findings imply that the impaired interaction of the VAN might explain higher risks of suicidal behaviors and executive function deficits related to victimization. As opposed to victimization, perpetration is negatively associated with functional connectivity between VAN and SMH. Back to the nature of the emergence of aggressive behaviors, according to the social information processing (SIP) model, the perpetrator’s aggressive behaviors might stem from cognitive distortions in the processing of social information, especially for the encoding of information [96–99]. However, the hostile pattern inherent in aggressive preadolescents would direct their attention to non-hostile cues that are inconsistent with the pattern, preventing further processing and recalling of such information, which could eventually lead to their aggressive behaviors [100]. The dynamic regulation of attention involved in this process requires the recruitment of multiple attentional networks, and VAN was considered to play a crucial role in detecting the pattern-relevant salient cues [101]. Thus, it is reasonable to infer that the negative association of the functional connectivity between VAN and SMH with perpetration might explain perpetrators’ particular “attention bias” (i.e., abnormal social information processing), thereby explaining their aggressive behaviors. The results of the current study provided a novel neurobiological basis for preadolescent victims and perpetrators underlying their behavioral mechanisms.

A few methodological limitations should be taken into consideration. Firstly, ABCD does not differentiate by specific age of bullying exposure, and only self-reported information about peer bullying was collected. Future studies should use more detailed assessments and consider incorporating information reported from other vital sources from various aspects of preadolescents’ life, such as peers, parents, and teachers. Secondly, although the American Community Survey post-stratification weights data in the wave of 2-year follow-up was not provided by ABCD, it should be considered in further analysis to avoid the biased estimates of sociodemographic characteristics of the sample. Thirdly, the associations of other meaningful variables like early life adversities and psychopathology (e.g., internalizing/externalizing problems) with peer bullying could be further explored in future studies [102, 103]. Finally, future research should incorporate more influential protective factors into the model to gain more accurate assessments of the impact of per-time bullying on preadolescents.

Despite these limitations, our study underscores that future interventions should consider the distinct psycho-cognitive patterns among bullying subtypes, and subdividing the target group into different subtypes may be necessary for enhancing the efficacy of traditional intervention programs. Specifically, cognitive training interventions that directly target executive function deficits may hold promise for addressing overt perpetration. Nevertheless, these approaches are unlikely to help preadolescents with relational perpetration due to their good executive function skills. Instead, interventions may need more effort into channeling their high cognitive skills for prosocial behaviors. Moreover, the differences among bullying subtypes should be considered to optimize anti-bullying intervention programs in schools and to benefit their early prevention. For instance, implementing a new curriculum (including videotapes and lectures) for students about the adverse impacts of bullying and its subtypes to promote attitudes against all forms of bullying and prosocial behaviors [104]. Meanwhile, training about corresponding handling strategies should also be given to teachers and other school staff to offer timely help and give extra attention to those who participated in bullying to reduce the adverse effects of the bullying experience on their suicidality/NSSI, neurocognition, and brain development.

## Conclusions

Overall, peer bullying had a pervasive effect on preadolescents’ suicidality/NSSI, cognitive functions, brain structure, and brain function. Meanwhile, different bullying subtypes presented distinct psycho-cognitive patterns among preadolescents. As the observations in the ABCD cohort continue to accumulate, the longitudinal effects of peer bullying and its subtypes on preadolescents’ behavioral- and neural- development could be further elucidated.

## Supplementary Information

**Additional file 1: Table S1.** ABCD data release 4.0 variables used in current analysis. **Figure S1.** Flowchart indicating exclusions for primary analyses. **Table S2.** Demographic characteristics of the analyzed samples. **Table S3.** Associations of peer bullying subtypes with suicidality/NSSI in preadolescents. **Table S4.** Associations of peer bullying subtypes with cognition in preadolescents. **Table S5.** Associations between peer bullying and brain structure. **Table S6.** Associations between peer bullying and brain network.

## Data Availability

The datasets used and/or analyzed during the current study are available from the corresponding author on reasonable request.

## Abbreviations

ABCD: Adolescent Brain Cognitive Development
AUN: Auditory network
BMI: Body mass index
CA: Cortical area
CON: Cingulo-opercular network
CPN: Cingulo-parietal network
CT: Cortical thickness
CV: Cortical volume
DAN: Dorsal attention network
DMN: Default mode network
FPN: Frontoparietal network
KSADS: Kiddle Schedule for Affective Disorders and Schizophrenia
NSSI: Non-suicidal self-injury
PEQ: Peer Experience Questionnaire
rsfMRI: Resting-state functional magnetic resonance imaging
RTN: Retrosplenial-temporal network
SA: Suicide attempt
SES: Socioeconomic status
SI: Suicide ideation
SMH: Sensorimotor hand network
SMM: Sensorimotor mouth network
sMRI: Structural magnetic resonance imaging
SN: Salience network
VAN: Ventral attention network
VN: Visual network
