# Supplementary material for: Associations of bullying perpetration and peer victimization subtypes with preadolescent’s suicidality, non-suicidal self-injury, neurocognition, and brain development
Source: BMC Med. 2023 Apr 12;21:141. doi: 10.1186/s12916-023-02808-8 (PMC10091581; doi:10.1186/s12916-023-02808-8)
Supplement: Supplementary file 1 — Additional file 1: Table S1. ABCD data release 4.0 variables used in current analysis. Figure S1. Flowchart indicating exclusions for primary analyses. Table S2. Demographic characteristics of the analyzed samples. Table S3. Associations of peer bullying subtypes with suicidality/NSSI in preadolescents. Table S4. Associations of peer bullying subtypes with cognition in preadolescents. Table S5. Associations between peer bullying and brain structure. Table S6. Associations between peer bullying and brain network. [file 12916_2023_2808_MOESM1_ESM.docx]

**Additional file 1**

**Table S1.** *ABCD data release 4.0 variables used in current analysis.*

| **Description** | **Field names** | **Source** |
| --- | --- | --- |
| **Sociodemographics** | |  |
| Sex at birth | demo_sex_v2 | pdem02 |
| Age | interview_age | pdem02 |
| Site | site_id_l | abcd_lt01 |
| Family_id | rel_family_id | acspsw03 |
| Race/Ethnicity | race_ethnicity | acspsw03 |
| Househould income | demo_comb_income_v2 | acspsw03 |
| Household marrital status | demo_prnt_marital_v2 | acspsw03 |
| Parental highest education | demo_prtnr_ed_v2; demo_prnt_ed_v2 | acspsw03 |
| BMI | anthroheightcalc; anthroweightcalc | abcd_ant_01 |
| Handedness | ehi_y_ss_scoreb | abcd_ehis01 |
| **Variables used in analysis** |  |  |
| Bullying perpetration/peer victimisation | . | abcd_peq01 |
| Child-reported SI, NSSI, SA | . | abcd_ksad501 |
| Emotion 2-back task (accuracy) | tfmri_nb_all_beh_c2b_rate | abcd_mrinback02 |
| Flanker task | nihtbx_flanker_agecorrected | abcd_tbss01 |
| Picture Sequence Memory task | nihtbx_picture_agecorrected | abcd_tbss01 |
| Pattern Comparison Processing Speed task | nihtbx_pattern_agecorrected | abcd_tbss01 |
| Brain structure (MRI) | . | abcd_mrisdp10201 |
| Quality control (MRI) | iqc_t1_ok_ser > 0 | mriqcrp10301 |
| Quality control (MRI) | fsqc_qc ~= 0 | abcd_fsurfqc01 |
| Quality control (MRI) | fsqc_qu_motion >1 | abcd_fsurfqc01 |
| Brain network (RSFC) | . | abcd_betnet02 |
| Quality control (RSFC) | rsfmri_c_ngd_meanmotion<0.25 | abcd_betnet02 |
| Quality control (RSFC) | iqc_rsfmri_good_ser>0 | mriqcrp10301 |

“.” means multiple variables were used in that scale.

**Figure s1.** *Flowchart indicating exclusions for primary analyses.*


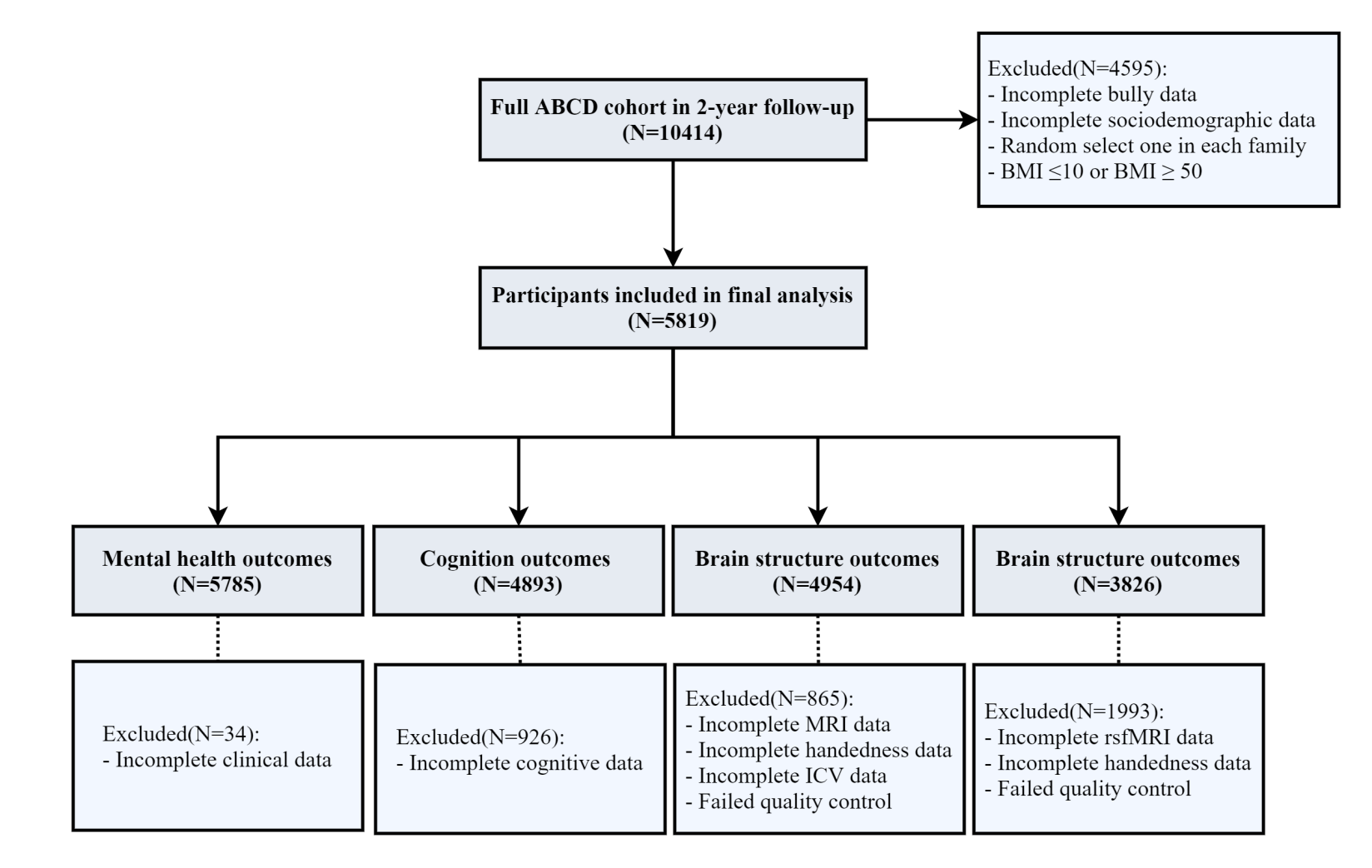


**Table S2.** *Demographic characteristics of the analyzed samples.*

|  | **Mental Health**  **(N=5785)** | **Cognition (N=4893)** | **MRI (N=4954)** | **rsfMRI (N=3826)** |  |
| --- | --- | --- | --- | --- | --- |
| **Characteristic** |  |  |  |  | ***P value*** |
| **Age, mean(SD), y** | 11.92 (0.6) | 11.89 (0.6) | 11.89 (0.6) | 11.91 (0.6) | 0.004 |
| **Sex** | | | | | 0.005 |
| Male | 3078 (53.2) | 2652 (54.2) | 2684 (54.2) | 1943 (50.8) |  |
| Female | 2707 (46.8) | 2241 (45.8) | 2270 (45.8) | 1883 (49.2) |  |
| **Race/ethnicity** | | | | | 0.902 |
| non-Hispanic White | 3324 (57.5) | 2791 (57.0) | 2809 (56.7) | 2235 (58.4) |  |
| non-Hispanic Black | 641 (11.1) | 563 (11.5) | 564 (11.4) | 395 (10.3) |  |
| Hispanic | 1116 (19.3) | 948 (19.4) | 973 (19.6) | 741 (19.4) |  |
| Asian | 128 ( 2.2) | 95 ( 1.9) | 100 ( 2.0) | 73 ( 1.9) |  |
| Other | 576 (10.0) | 496 (10.1) | 508 (10.3) | 382 (10.0) |  |
| **Household married** | | | | | 0.534 |
| Married | 4117 (71.2) | 3462 (70.8) | 3511 (70.9) | 2758 (72.1) |  |
| Unmarried | 1668 (28.8) | 1431 (29.2) | 1443 (29.1) | 1068 (27.9) |  |
| **Household income** | | | | | 0.403 |
| <50k | 1580 (27.3) | 1384 (28.3) | 1404 (28.3) | 1024 (26.8) |  |
| ≥50k&<100k | 1709 (29.5) | 1473 (30.1) | 1491 (30.1) | 1177 (30.8) |  |
| ≥100k | 2496 (43.2) | 2036 (41.6) | 2059 (41.6) | 1625 (42.5) |  |
| **Parental highest attained education level**a | | | | | 0.827 |
| Below High School | 210 ( 3.6) | 180 ( 3.7) | 184 ( 3.7) | 131 ( 3.4) |  |
| High School Grad/GED | 422 ( 7.3) | 375 ( 7.7) | 378 ( 7.6) | 264 ( 6.9) |  |
| Some College | 1403 (24.3) | 1232 (25.2) | 1251 (25.3) | 926 (24.2) |  |
| Bachelor Degree | 1585 (27.4) | 1307 (26.7) | 1343 (27.1) | 1047 (27.4) |  |
| Postgraduate Degree | 2165 (37.4) | 1799 (36.8) | 1798 (36.3) | 1458 (38.1) |  |
| **BMI, mean(SD)** | 20.6 (4.8) | 20.6 (4.8) | 20.6 (4.8) | 20.3 (4.6) | 0.007 |
|  | | | | |  |

**Table S3.** *Associations of peer bullying subtypes with suicidality/NSSI in preadolescents.*

| **Predictor** |  | **95 CI** | |  |
| --- | --- | --- | --- | --- |
|  | **Estimate** | **Lower** | **Upper** | ***P* value** |
| **Suicide ideation** |  |  |  |  |
| Overt victimization | 1.253 | 1.132 | 1.386 | < .001 |
| Relational victimization | 1.406 | 1.263 | 1.565 | < .001 |
| Reputation victimization | 1.171 | 1.054 | 1.300 | 0.003 |
| Overt perpetration | 1.148 | 1.051 | 1.254 | 0.002 |
| Relational perpetration | 1.099 | 0.993 | 1.217 | 0.067 |
| Reputation perpetration | 1.021 | 0.943 | 1.106 | 0.600 |
| **Non-suicidal self-injury** |  |  |  |  |
| Overt victimization  Relational victimization  Reputation victimization | 1.497 | 1.326 | 1.690 | < .001 |
|  | 1.088 | 0.944 | 1.254 | 0.245 |
|  | 1.142 | 0.997 | 1.307 | 0.055 |
| Overt perpetration  Relational perpetration  Reputation perpetration | 1.049 | 0.941 | 1.168 | 0.389 |
|  | 1.183 | 1.043 | 1.342 | 0.009 |
|  | 1.095 | 1.000 | 1.199 | 0.051 |
| **Suicide attempt** |  |  |  |  |
| Overt victimization  Relational victimization  Reputation victimization | 1.226 | 1.005 | 1.495 | 0.044 |
|  | 1.310 | 1.037 | 1.653 | 0.023 |
|  | 1.224 | 0.978 | 1.533 | 0.077 |
| Overt perpetration  Relational perpetration  Reputation perpetration | 1.231 | 1.054 | 1.438 | 0.009 |
|  | 1.142 | 0.925 | 1.411 | 0.218 |
|  | 0.960 | 0.824 | 1.117 | 0.596 |
| Odds radio was reported for binary suicidality/NSSI variables. | | | | |
| Adjusted for age, sex, race/ethnicity, site, BMI, marital status, income, and parental highest education. | | | | |
|  |  |  |  |  |

|  |
| --- |
|  |

**Table S4.** *Associations of peer bullying subtypes with cognition in preadolescents.*

| **Predictor** |  | **95 CI** | |  |
| --- | --- | --- | --- | --- |
|  | **Estimate** | **Lower** | **Upper** | ***P* value** |
| **Emotion 2-back task** |  |  |  |  |
| Overt victimization | -0.012 | -0.047 | 0.022 | 0.483 |
| Relational victimization | 0.028 | -0.006 | 0.061 | 0.106 |
| Reputation victimization | -0.036 | -0.071 | -0.001 | 0.044 |
| Overt perpetration | -0.037 | -0.069 | -0.006 | 0.021 |
| Relational perpetration | 0.069 | 0.038 | 0.100 | < .001 |
| Reputation perpetration | -0.020 | -0.050 | 0.010 | 0.185 |
| **Picture Sequence Memory task** |  |  |  |  |
| Overt victimization  Relational victimization  Reputation victimization | -0.017 | -0.053 | 0.019 | 0.353 |
|  | 0.009 | -0.026 | 0.043 | 0.625 |
|  | -0.044 | -0.080 | -0.008 | 0.017 |
| Overt perpetration  Relational perpetration  Reputation perpetration | -0.013 | -0.046 | 0.020 | 0.429 |
|  | 0.055 | 0.024 | 0.087 | 0.001 |
|  | -0.037 | -0.068 | -0.007 | 0.017 |
| **Flanker task** |  |  |  |  |
| Overt victimization  Relational victimization  Reputation victimization | 0.001 | -0.036 | 0.037 | 0.973 |
|  | 0.018 | -0.018 | 0.053 | 0.325 |
|  | -0.023 | -0.060 | 0.014 | 0.219 |
| Overt perpetration  Relational perpetration  Reputation perpetration | -0.036 | -0.069 | -0.002 | 0.037 |
|  | 0.072 | 0.040 | 0.105 | < .001 |
|  | 0.006 | -0.025 | 0.038 | 0.689 |
| **Pattern Comparison Processing Speed task** |  |  |  |  |
| Overt victimization | 0.000 | -0.037 | 0.036 | 0.997 |
| Relational victimization | 0.020 | -0.016 | 0.055 | 0.274 |
| Reputation victimization | -0.033 | -0.070 | 0.004 | 0.080 |
| Overt perpetration | -0.021 | -0.055 | 0.012 | 0.211 |
| Relational perpetration | 0.042 | 0.010 | 0.074 | 0.010 |
| Reputation perpetration | -0.017 | -0.048 | 0.015 | 0.299 |
| β coefficient was reported for continuous neurocognition variables; | | | | |
| Adjusted for age, sex, race/ethnicity, site, BMI, marital status, income, and parental highest education. | | | | |

# **Table S5**. *Associations between peer bullying and brain structure.*

| **sMRI** | **Victimisation** | |  | **Perpetration** | |  |
| --- | --- | --- | --- | --- | --- | --- |
|  | ***β*** | ***p_fdr_*** |  | ***β*** | ***p_fdr_*** | ***R^2^*** |
| CA.G_and_S_cingul-Ant | -0.007 | 0.760 |  | 0.014 | 0.996 | 0.612 |
| CA.G_and_S_cingul-Mid-Ant | -0.009 | 0.760 |  | 0.021 | 0.996 | 0.485 |
| CA.G_and_S_cingul-Mid-Post | -0.013 | 0.760 |  | 0.010 | 0.996 | 0.468 |
| CA.G_and_S_frontomargin | -0.014 | 0.760 |  | 0.008 | 0.996 | 0.444 |
| CA.G_and_S_occipital_inf | 0.015 | 0.760 |  | -0.015 | 0.996 | 0.368 |
| CA.G_and_S_paracentral | 0.018 | 0.760 |  | -0.003 | 0.996 | 0.380 |
| CA.G_and_S_subcentral | 0.000 | 0.993 |  | 0.009 | 0.996 | 0.480 |
| CA.G_and_S_transv_frontopol | -0.004 | 0.828 |  | -0.004 | 0.996 | 0.405 |
| CA.G_cingul-Post-dorsal | 0.044 | 0.045 |  | -0.022 | 0.996 | 0.434 |
| CA.G_cingul-Post-ventral | 0.013 | 0.760 |  | -0.010 | 0.996 | 0.241 |
| CA.G_cuneus | 0.023 | 0.760 |  | -0.018 | 0.996 | 0.265 |
| CA.G_front_inf-Opercular | 0.020 | 0.760 |  | -0.020 | 0.996 | 0.318 |
| CA.G_front_inf-Orbital | 0.027 | 0.760 |  | -0.032 | 0.713 | 0.219 |
| CA.G_front_inf-Triangul | 0.028 | 0.760 |  | -0.006 | 0.996 | 0.227 |
| CA.G_front_middle | -0.009 | 0.760 |  | 0.002 | 0.996 | 0.490 |
| CA.G_front_sup | 0.001 | 0.974 |  | 0.003 | 0.996 | 0.586 |
| CA.G_Ins_lg_and_S_cent_ins | -0.001 | 0.974 |  | -0.006 | 0.996 | 0.436 |
| CA.G_insular_short | -0.008 | 0.760 |  | -0.008 | 0.996 | 0.430 |
| CA.G_oc-temp_lat-fusifor | -0.008 | 0.760 |  | 0.001 | 0.996 | 0.417 |
| CA.G_oc-temp_med-Lingual | 0.011 | 0.760 |  | -0.011 | 0.996 | 0.226 |
| CA.G_oc-temp_med-Parahip | -0.006 | 0.828 |  | 0.004 | 0.996 | 0.206 |
| CA.G_occipital_middle | -0.011 | 0.760 |  | 0.008 | 0.996 | 0.417 |
| CA.G_occipital_sup | 0.015 | 0.760 |  | -0.017 | 0.996 | 0.366 |
| CA.G_orbital | -0.002 | 0.894 |  | 0.000 | 0.996 | 0.585 |
| CA.G_pariet_inf-Angular | -0.016 | 0.760 |  | 0.004 | 0.996 | 0.387 |
| CA.G_pariet_inf-Supramar | -0.005 | 0.828 |  | 0.019 | 0.996 | 0.433 |
| CA.G_parietal_sup | 0.007 | 0.781 |  | 0.005 | 0.996 | 0.363 |
| CA.G_postcentral | -0.004 | 0.828 |  | -0.001 | 0.996 | 0.546 |
| CA.G_precentral | 0.004 | 0.828 |  | -0.001 | 0.996 | 0.539 |
| CA.G_precuneus | 0.011 | 0.760 |  | -0.010 | 0.996 | 0.419 |
| CA.G_rectus | -0.002 | 0.894 |  | 0.008 | 0.996 | 0.570 |
| CA.G_subcallosal | -0.012 | 0.760 |  | -0.011 | 0.996 | 0.345 |
| CA.G_temp_sup-G_T_transv | 0.014 | 0.760 |  | -0.001 | 0.996 | 0.268 |
| CA.G_temp_sup-Lateral | 0.024 | 0.760 |  | -0.012 | 0.996 | 0.564 |
| CA.G_temp_sup-Plan_polar | 0.017 | 0.760 |  | -0.005 | 0.996 | 0.447 |
| CA.G_temp_sup-Plan_tempo | 0.020 | 0.760 |  | -0.031 | 0.713 | 0.352 |
| CA.G_temporal_inf | -0.012 | 0.760 |  | 0.018 | 0.996 | 0.494 |
| CA.G_temporal_middle | -0.008 | 0.760 |  | 0.005 | 0.996 | 0.538 |
| CA.Lat_Fis-ant-Horizont | 0.024 | 0.760 |  | -0.004 | 0.996 | 0.231 |
| CA.Lat_Fis-ant-Vertical | 0.013 | 0.760 |  | 0.008 | 0.996 | 0.134 |
| CA.Lat_Fis-post | 0.024 | 0.760 |  | -0.009 | 0.996 | 0.339 |
| CA.Pole_occipital | 0.010 | 0.760 |  | -0.007 | 0.996 | 0.382 |
| CA.Pole_temporal | 0.004 | 0.828 |  | -0.007 | 0.996 | 0.452 |
| CA.S_calcarine | 0.008 | 0.781 |  | -0.004 | 0.996 | 0.178 |
| CA.S_central | 0.007 | 0.760 |  | -0.021 | 0.996 | 0.491 |
| CA.S_cingul-Marginalis | 0.004 | 0.828 |  | -0.010 | 0.996 | 0.387 |
| CA.S_circular_insula_ant | -0.008 | 0.760 |  | -0.006 | 0.996 | 0.309 |
| CA.S_circular_insula_inf | 0.012 | 0.760 |  | 0.009 | 0.996 | 0.428 |
| CA.S_circular_insula_sup | 0.016 | 0.760 |  | -0.003 | 0.996 | 0.460 |
| CA.S_collat_transv_ant | -0.014 | 0.760 |  | 0.001 | 0.996 | 0.283 |
| CA.S_collat_transv_post | 0.009 | 0.760 |  | -0.024 | 0.996 | 0.151 |
| CA.S_front_inf | 0.010 | 0.760 |  | -0.001 | 0.996 | 0.418 |
| CA.S_front_middle | -0.025 | 0.760 |  | 0.007 | 0.996 | 0.398 |
| CA.S_front_sup | -0.011 | 0.760 |  | -0.029 | 0.713 | 0.465 |
| CA.S_interm_prim-Jensen | -0.005 | 0.828 |  | 0.006 | 0.996 | 0.136 |
| CA.S_intrapariet_and_P_trans | -0.019 | 0.760 |  | 0.008 | 0.996 | 0.384 |
| CA.S_oc_middle_and_Lunatus | -0.010 | 0.760 |  | -0.007 | 0.996 | 0.251 |
| CA.S_oc_sup_and_transversal | -0.010 | 0.760 |  | -0.001 | 0.996 | 0.353 |
| CA.S_oc-temp_lat | 0.008 | 0.760 |  | 0.003 | 0.996 | 0.430 |
| CA.S_oc-temp_med_and_Lingual | -0.016 | 0.760 |  | 0.000 | 0.996 | 0.446 |
| CA.S_occipital_ant | 0.000 | 0.993 |  | 0.020 | 0.996 | 0.273 |
| CA.S_orbital_lateral | 0.006 | 0.828 |  | 0.007 | 0.996 | 0.248 |
| CA.S_orbital_med-olfact | -0.013 | 0.760 |  | 0.012 | 0.996 | 0.450 |
| CA.S_orbital-H_Shaped | -0.005 | 0.828 |  | 0.001 | 0.996 | 0.496 |
| CA.S_parieto_occipital | 0.007 | 0.781 |  | 0.005 | 0.996 | 0.355 |
| CA.S_pericallosal | -0.009 | 0.760 |  | -0.008 | 0.996 | 0.474 |
| CA.S_postcentral | 0.010 | 0.760 |  | 0.008 | 0.996 | 0.425 |
| CA.S_precentral-inf-part | -0.013 | 0.760 |  | 0.006 | 0.996 | 0.366 |
| CA.S_precentral-sup-part | 0.010 | 0.760 |  | -0.006 | 0.996 | 0.297 |
| CA.S_suborbital | -0.013 | 0.760 |  | 0.014 | 0.996 | 0.271 |
| CA.S_subparietal | 0.016 | 0.760 |  | 0.000 | 0.996 | 0.337 |
| CA.S_temporal_inf | -0.020 | 0.760 |  | 0.023 | 0.996 | 0.459 |
| CA.S_temporal_sup | -0.007 | 0.760 |  | 0.005 | 0.996 | 0.549 |
| CA.S_temporal_transverse | 0.030 | 0.760 |  | 0.000 | 0.996 | 0.251 |
| CT.G_and_S_cingul-Ant | 0.007 | 0.944 |  | 0.000 | 0.976 | 0.099 |
| CT.G_and_S_cingul-Mid-Ant | -0.027 | 0.619 |  | 0.007 | 0.909 | 0.104 |
| CT.G_and_S_cingul-Mid-Post | -0.016 | 0.770 |  | -0.005 | 0.909 | 0.082 |
| CT.G_and_S_frontomargin | 0.023 | 0.619 |  | -0.021 | 0.909 | 0.077 |
| CT.G_and_S_occipital_inf | -0.019 | 0.648 |  | -0.013 | 0.909 | 0.084 |
| CT.G_and_S_paracentral | -0.052 | 0.047 |  | 0.010 | 0.909 | 0.140 |
| CT.G_and_S_subcentral | -0.001 | 0.962 |  | -0.018 | 0.909 | 0.053 |
| CT.G_and_S_transv_frontopol | 0.018 | 0.678 |  | -0.005 | 0.909 | 0.080 |
| CT.G_cingul-Post-dorsal | -0.006 | 0.956 |  | 0.002 | 0.959 | 0.053 |
| CT.G_cingul-Post-ventral | 0.012 | 0.858 |  | -0.021 | 0.909 | 0.058 |
| CT.G_cuneus | -0.011 | 0.858 |  | -0.008 | 0.909 | 0.134 |
| CT.G_front_inf-Opercular | -0.012 | 0.858 |  | 0.013 | 0.909 | 0.051 |
| CT.G_front_inf-Orbital | -0.001 | 0.968 |  | 0.009 | 0.909 | 0.029 |
| CT.G_front_inf-Triangul | 0.013 | 0.858 |  | -0.012 | 0.909 | 0.039 |
| CT.G_front_middle | -0.002 | 0.962 |  | 0.005 | 0.909 | 0.062 |
| CT.G_front_sup | -0.024 | 0.619 |  | 0.013 | 0.909 | 0.077 |
| CT.G_Ins_lg_and_S_cent_ins | 0.017 | 0.722 |  | 0.016 | 0.909 | 0.070 |
| CT.G_insular_short | -0.011 | 0.858 |  | 0.019 | 0.909 | 0.090 |
| CT.G_oc-temp_lat-fusifor | -0.019 | 0.648 |  | -0.021 | 0.909 | 0.068 |
| CT.G_oc-temp_med-Lingual | 0.001 | 0.962 |  | -0.013 | 0.909 | 0.229 |
| CT.G_oc-temp_med-Parahip | -0.011 | 0.858 |  | 0.008 | 0.909 | 0.059 |
| CT.G_occipital_middle | -0.012 | 0.858 |  | -0.003 | 0.950 | 0.166 |
| CT.G_occipital_sup | -0.018 | 0.648 |  | -0.005 | 0.909 | 0.110 |
| CT.G_orbital | -0.002 | 0.962 |  | -0.010 | 0.909 | 0.058 |
| CT.G_pariet_inf-Angular | -0.025 | 0.619 |  | 0.003 | 0.950 | 0.084 |
| CT.G_pariet_inf-Supramar | -0.021 | 0.626 |  | -0.006 | 0.909 | 0.135 |
| CT.G_parietal_sup | -0.032 | 0.619 |  | -0.005 | 0.909 | 0.086 |
| CT.G_postcentral | -0.002 | 0.962 |  | -0.029 | 0.909 | 0.168 |
| CT.G_precentral | -0.033 | 0.619 |  | 0.020 | 0.909 | 0.110 |
| CT.G_precuneus | -0.025 | 0.619 |  | 0.005 | 0.909 | 0.059 |
| CT.G_rectus | -0.006 | 0.944 |  | -0.015 | 0.909 | 0.084 |
| CT.G_subcallosal | 0.017 | 0.678 |  | -0.006 | 0.909 | 0.184 |
| CT.G_temp_sup-G_T_transv | -0.005 | 0.956 |  | 0.008 | 0.909 | 0.049 |
| CT.G_temp_sup-Lateral | -0.012 | 0.858 |  | 0.008 | 0.909 | 0.078 |
| CT.G_temp_sup-Plan_polar | -0.024 | 0.619 |  | 0.014 | 0.909 | 0.070 |
| CT.G_temp_sup-Plan_tempo | -0.019 | 0.648 |  | -0.006 | 0.909 | 0.042 |
| CT.G_temporal_inf | 0.009 | 0.870 |  | -0.018 | 0.909 | 0.076 |
| CT.G_temporal_middle | -0.011 | 0.858 |  | 0.006 | 0.909 | 0.136 |
| CT.Lat_Fis-ant-Horizont | 0.001 | 0.962 |  | 0.006 | 0.909 | 0.036 |
| CT.Lat_Fis-ant-Vertical | 0.002 | 0.962 |  | 0.015 | 0.909 | 0.039 |
| CT.Lat_Fis-post | -0.002 | 0.962 |  | -0.011 | 0.909 | 0.076 |
| CT.Pole_occipital | -0.024 | 0.619 |  | 0.010 | 0.909 | 0.374 |
| CT.Pole_temporal | -0.021 | 0.626 |  | -0.003 | 0.950 | 0.040 |
| CT.S_calcarine | 0.002 | 0.962 |  | -0.006 | 0.909 | 0.165 |
| CT.S_central | -0.029 | 0.619 |  | -0.018 | 0.909 | 0.106 |
| CT.S_cingul-Marginalis | -0.023 | 0.619 |  | -0.001 | 0.959 | 0.117 |
| CT.S_circular_insula_ant | -0.007 | 0.944 |  | 0.009 | 0.909 | 0.080 |
| CT.S_circular_insula_inf | 0.005 | 0.956 |  | 0.001 | 0.959 | 0.096 |
| CT.S_circular_insula_sup | -0.020 | 0.626 |  | 0.009 | 0.909 | 0.131 |
| CT.S_collat_transv_ant | 0.010 | 0.858 |  | -0.007 | 0.909 | 0.044 |
| CT.S_collat_transv_post | -0.022 | 0.619 |  | -0.012 | 0.909 | 0.073 |
| CT.S_front_inf | 0.024 | 0.619 |  | -0.021 | 0.909 | 0.118 |
| CT.S_front_middle | 0.002 | 0.962 |  | 0.011 | 0.909 | 0.139 |
| CT.S_front_sup | -0.010 | 0.858 |  | 0.008 | 0.909 | 0.149 |
| CT.S_interm_prim-Jensen | 0.010 | 0.858 |  | -0.002 | 0.950 | 0.035 |
| CT.S_intrapariet_and_P_trans | -0.024 | 0.619 |  | -0.005 | 0.909 | 0.090 |
| CT.S_oc_middle_and_Lunatus | -0.010 | 0.858 |  | -0.033 | 0.909 | 0.167 |
| CT.S_oc_sup_and_transversal | -0.002 | 0.962 |  | -0.009 | 0.909 | 0.097 |
| CT.S_oc-temp_lat | 0.013 | 0.858 |  | -0.010 | 0.909 | 0.085 |
| CT.S_oc-temp_med_and_Lingual | -0.009 | 0.858 |  | -0.013 | 0.909 | 0.118 |
| CT.S_occipital_ant | 0.002 | 0.962 |  | -0.028 | 0.909 | 0.101 |
| CT.S_orbital_lateral | 0.022 | 0.619 |  | -0.022 | 0.909 | 0.060 |
| CT.S_orbital_med-olfact | -0.004 | 0.962 |  | 0.014 | 0.909 | 0.087 |
| CT.S_orbital-H_Shaped | 0.003 | 0.962 |  | -0.022 | 0.909 | 0.084 |
| CT.S_parieto_occipital | -0.002 | 0.962 |  | -0.029 | 0.909 | 0.118 |
| CT.S_pericallosal | -0.023 | 0.619 |  | 0.023 | 0.909 | 0.035 |
| CT.S_postcentral | -0.014 | 0.858 |  | -0.016 | 0.909 | 0.129 |
| CT.S_precentral-inf-part | -0.008 | 0.890 |  | -0.002 | 0.950 | 0.064 |
| CT.S_precentral-sup-part | -0.033 | 0.619 |  | 0.046 | 0.301 | 0.081 |
| CT.S_suborbital | -0.006 | 0.944 |  | -0.003 | 0.950 | 0.081 |
| CT.S_subparietal | -0.023 | 0.619 |  | -0.006 | 0.909 | 0.106 |
| CT.S_temporal_inf | 0.002 | 0.962 |  | -0.016 | 0.909 | 0.117 |
| CT.S_temporal_sup | -0.009 | 0.858 |  | -0.011 | 0.909 | 0.108 |
| CT.S_temporal_transverse | -0.021 | 0.626 |  | 0.004 | 0.950 | 0.029 |
| CV.G_and_S_cingul-Ant | -0.010 | 0.711 |  | 0.011 | 0.990 | 0.597 |
| CV.G_and_S_cingul-Mid-Ant | -0.021 | 0.711 |  | 0.022 | 0.990 | 0.463 |
| CV.G_and_S_cingul-Mid-Post | -0.015 | 0.711 |  | 0.007 | 0.990 | 0.467 |
| CV.G_and_S_frontomargin | -0.002 | 0.956 |  | -0.003 | 0.990 | 0.371 |
| CV.G_and_S_occipital_inf | 0.002 | 0.956 |  | -0.017 | 0.990 | 0.347 |
| CV.G_and_S_paracentral | -0.019 | 0.711 |  | 0.003 | 0.990 | 0.286 |
| CV.G_and_S_subcentral | 0.000 | 0.989 |  | 0.002 | 0.990 | 0.472 |
| CV.G_and_S_transv_frontopol | 0.000 | 0.989 |  | 0.002 | 0.990 | 0.334 |
| CV.G_cingul-Post-dorsal | 0.039 | 0.144 |  | -0.017 | 0.990 | 0.453 |
| CV.G_cingul-Post-ventral | 0.017 | 0.711 |  | -0.020 | 0.990 | 0.234 |
| CV.G_cuneus | 0.019 | 0.711 |  | -0.022 | 0.990 | 0.271 |
| CV.G_front_inf-Opercular | 0.013 | 0.711 |  | -0.009 | 0.990 | 0.321 |
| CV.G_front_inf-Orbital | 0.021 | 0.711 |  | -0.024 | 0.990 | 0.229 |
| CV.G_front_inf-Triangul | 0.033 | 0.703 |  | -0.009 | 0.990 | 0.206 |
| CV.G_front_middle | -0.013 | 0.711 |  | 0.004 | 0.990 | 0.486 |
| CV.G_front_sup | -0.016 | 0.711 |  | 0.011 | 0.990 | 0.595 |
| CV.G_Ins_lg_and_S_cent_ins | 0.001 | 0.989 |  | 0.009 | 0.990 | 0.431 |
| CV.G_insular_short | -0.016 | 0.711 |  | -0.002 | 0.990 | 0.435 |
| CV.G_oc-temp_lat-fusifor | -0.012 | 0.711 |  | -0.006 | 0.990 | 0.440 |
| CV.G_oc-temp_med-Lingual | 0.008 | 0.833 |  | -0.018 | 0.990 | 0.300 |
| CV.G_oc-temp_med-Parahip | -0.008 | 0.833 |  | 0.009 | 0.990 | 0.177 |
| CV.G_occipital_middle | -0.018 | 0.711 |  | 0.008 | 0.990 | 0.391 |
| CV.G_occipital_sup | 0.000 | 0.989 |  | -0.016 | 0.990 | 0.317 |
| CV.G_orbital | -0.003 | 0.935 |  | -0.001 | 0.990 | 0.567 |
| CV.G_pariet_inf-Angular | -0.030 | 0.703 |  | 0.006 | 0.990 | 0.352 |
| CV.G_pariet_inf-Supramar | -0.017 | 0.711 |  | 0.018 | 0.990 | 0.414 |
| CV.G_parietal_sup | -0.010 | 0.745 |  | 0.001 | 0.995 | 0.347 |
| CV.G_postcentral | -0.010 | 0.745 |  | -0.017 | 0.990 | 0.371 |
| CV.G_precentral | -0.022 | 0.711 |  | 0.008 | 0.990 | 0.458 |
| CV.G_precuneus | 0.002 | 0.938 |  | -0.009 | 0.990 | 0.440 |
| CV.G_rectus | -0.003 | 0.938 |  | 0.001 | 0.995 | 0.515 |
| CV.G_subcallosal | -0.003 | 0.938 |  | -0.012 | 0.990 | 0.378 |
| CV.G_temp_sup-G_T_transv | 0.013 | 0.711 |  | 0.005 | 0.990 | 0.292 |
| CV.G_temp_sup-Lateral | 0.014 | 0.711 |  | -0.002 | 0.990 | 0.468 |
| CV.G_temp_sup-Plan_polar | 0.007 | 0.833 |  | 0.007 | 0.990 | 0.460 |
| CV.G_temp_sup-Plan_tempo | 0.014 | 0.711 |  | -0.033 | 0.990 | 0.319 |
| CV.G_temporal_inf | -0.006 | 0.833 |  | 0.010 | 0.990 | 0.506 |
| CV.G_temporal_middle | -0.014 | 0.711 |  | 0.010 | 0.990 | 0.516 |
| CV.Lat_Fis-ant-Horizont | 0.021 | 0.711 |  | -0.002 | 0.990 | 0.165 |
| CV.Lat_Fis-ant-Vertical | 0.011 | 0.745 |  | 0.012 | 0.990 | 0.155 |
| CV.Lat_Fis-post | 0.026 | 0.711 |  | -0.018 | 0.990 | 0.320 |
| CV.Pole_occipital | -0.010 | 0.711 |  | 0.003 | 0.990 | 0.398 |
| CV.Pole_temporal | -0.007 | 0.833 |  | -0.004 | 0.990 | 0.412 |
| CV.S_calcarine | 0.007 | 0.833 |  | -0.006 | 0.990 | 0.230 |
| CV.S_central | -0.004 | 0.927 |  | -0.026 | 0.990 | 0.434 |
| CV.S_cingul-Marginalis | 0.005 | 0.885 |  | -0.015 | 0.990 | 0.395 |
| CV.S_circular_insula_ant | -0.011 | 0.711 |  | -0.001 | 0.990 | 0.374 |
| CV.S_circular_insula_inf | 0.011 | 0.711 |  | 0.010 | 0.990 | 0.400 |
| CV.S_circular_insula_sup | 0.010 | 0.711 |  | 0.003 | 0.990 | 0.459 |
| CV.S_collat_transv_ant | -0.013 | 0.711 |  | -0.006 | 0.990 | 0.387 |
| CV.S_collat_transv_post | -0.004 | 0.935 |  | -0.024 | 0.990 | 0.150 |
| CV.S_front_inf | 0.019 | 0.711 |  | -0.005 | 0.990 | 0.380 |
| CV.S_front_middle | -0.017 | 0.711 |  | 0.006 | 0.990 | 0.331 |
| CV.S_front_sup | -0.014 | 0.711 |  | -0.028 | 0.990 | 0.428 |
| CV.S_interm_prim-Jensen | -0.006 | 0.881 |  | 0.005 | 0.990 | 0.152 |
| CV.S_intrapariet_and_P_trans | -0.026 | 0.711 |  | 0.008 | 0.990 | 0.392 |
| CV.S_oc_middle_and_Lunatus | -0.010 | 0.745 |  | -0.016 | 0.990 | 0.261 |
| CV.S_oc_sup_and_transversal | -0.012 | 0.711 |  | -0.004 | 0.990 | 0.360 |
| CV.S_oc-temp_lat | 0.009 | 0.745 |  | 0.002 | 0.990 | 0.462 |
| CV.S_oc-temp_med_and_Lingual | -0.020 | 0.711 |  | -0.003 | 0.990 | 0.494 |
| CV.S_occipital_ant | 0.000 | 0.989 |  | 0.013 | 0.990 | 0.303 |
| CV.S_orbital_lateral | 0.015 | 0.711 |  | -0.004 | 0.990 | 0.197 |
| CV.S_orbital_med-olfact | -0.011 | 0.711 |  | 0.014 | 0.990 | 0.495 |
| CV.S_orbital-H_Shaped | -0.003 | 0.935 |  | -0.006 | 0.990 | 0.482 |
| CV.S_parieto_occipital | 0.006 | 0.833 |  | -0.003 | 0.990 | 0.368 |
| CV.S_pericallosal | -0.018 | 0.711 |  | 0.005 | 0.990 | 0.379 |
| CV.S_postcentral | 0.012 | 0.711 |  | 0.000 | 0.997 | 0.422 |
| CV.S_precentral-inf-part | -0.015 | 0.711 |  | 0.003 | 0.990 | 0.336 |
| CV.S_precentral-sup-part | 0.003 | 0.938 |  | 0.005 | 0.990 | 0.297 |
| CV.S_suborbital | -0.016 | 0.711 |  | 0.019 | 0.990 | 0.165 |
| CV.S_subparietal | 0.005 | 0.885 |  | -0.001 | 0.994 | 0.348 |
| CV.S_temporal_inf | -0.015 | 0.711 |  | 0.015 | 0.990 | 0.480 |
| CV.S_temporal_sup | -0.007 | 0.790 |  | 0.000 | 0.997 | 0.547 |
| CV.S_temporal_transverse | 0.023 | 0.711 |  | 0.003 | 0.990 | 0.193 |

Adjusted for age, sex, race/ethnicity, site, BMI, marital status, income, parental highest education, intracranial volume (ICV) and handedness.

# **Table S6.** *Associations between peer bullying and brain network.*

| **rsfMRI** | **Victimisation** | |  | **Perpetration** | | ***R^2^*** |
| --- | --- | --- | --- | --- | --- | --- |
|  | ***β*** | ***p_fdr_*** |  | ***β*** | ***p_fdr_*** |  |
| AUN_AUN | 0.005 | 0.771 |  | 0.005 | 0.882 | 0.040 |
| CPN_CPN | 0.013 | 0.720 |  | -0.017 | 0.854 | 0.046 |
| CON_CON | -0.019 | 0.685 |  | 0.019 | 0.854 | 0.111 |
| DAN_DAN | -0.032 | 0.471 |  | 0.033 | 0.812 | 0.051 |
| DMN_DMN | -0.027 | 0.471 |  | -0.004 | 0.882 | 0.183 |
| FPN_FPN | -0.009 | 0.720 |  | 0.002 | 0.913 | 0.093 |
| RTN_RTN | 0.019 | 0.685 |  | -0.007 | 0.882 | 0.086 |
| SN_SN | 0.011 | 0.720 |  | -0.013 | 0.854 | 0.039 |
| SMH_SMH | 0.027 | 0.471 |  | 0.012 | 0.854 | 0.134 |
| SMM_SMM | 0.012 | 0.720 |  | 0.010 | 0.874 | 0.050 |
| VN_VN | 0.016 | 0.685 |  | -0.012 | 0.854 | 0.211 |
| VAN_VAN | -0.008 | 0.726 |  | 0.018 | 0.854 | 0.044 |
| AUN_DMN | 0.002 | 0.929 |  | 0.003 | 0.964 | 0.093 |
| CPN_DMN | -0.034 | 0.149 |  | -0.012 | 0.835 | 0.021 |
| CON_DMN | 0.034 | 0.137 |  | -0.016 | 0.667 | 0.144 |
| DMN_DAN | 0.048 | 0.022 |  | -0.016 | 0.667 | 0.173 |
| DMN_FPN | 0.050 | 0.024 |  | 0.005 | 0.964 | 0.046 |
| DMN_RTN | -0.025 | 0.332 |  | 0.001 | 0.964 | 0.021 |
| DMN_SN | 0.015 | 0.653 |  | -0.018 | 0.667 | 0.023 |
| DMN_SMH | 0.004 | 0.893 |  | -0.033 | 0.388 | 0.051 |
| DMN_SMM | -0.008 | 0.881 |  | 0.025 | 0.651 | 0.078 |
| DMN_VN | 0.005 | 0.893 |  | -0.003 | 0.964 | 0.132 |
| DMN_VAN | -0.065 | 0.004 |  | 0.034 | 0.388 | 0.060 |
| AUN_FPN | -0.008 | 0.818 |  | 0.000 | 0.992 | 0.079 |
| CPN_FPN | -0.009 | 0.818 |  | 0.005 | 0.980 | 0.015 |
| CON_FPN | -0.002 | 0.926 |  | -0.003 | 0.980 | 0.044 |
| DAN_FPN | -0.020 | 0.818 |  | 0.002 | 0.980 | 0.089 |
| DMN_FPN | 0.050 | 0.065 |  | 0.005 | 0.980 | 0.046 |
| FPN_RTN | 0.008 | 0.818 |  | 0.016 | 0.980 | 0.043 |
| FPN_SN | -0.010 | 0.818 |  | 0.016 | 0.980 | 0.037 |
| FPN_SMH | -0.008 | 0.818 |  | -0.027 | 0.844 | 0.063 |
| FPN_SMM | -0.016 | 0.818 |  | 0.014 | 0.980 | 0.045 |
| FPN_VN | -0.045 | 0.065 |  | 0.025 | 0.844 | 0.111 |
| FPN_VAN | 0.005 | 0.845 |  | -0.004 | 0.980 | 0.047 |
| AUN_SMH | 0.015 | 0.869 |  | 0.000 | 0.998 | 0.068 |
| CPN_SMH | -0.032 | 0.432 |  | 0.059 | 0.015 | 0.053 |
| CON_SMH | -0.020 | 0.720 |  | 0.053 | 0.015 | 0.058 |
| DAN_SMH | 0.005 | 0.893 |  | 0.022 | 0.394 | 0.028 |
| DMN_SMH | 0.004 | 0.893 |  | -0.033 | 0.194 | 0.051 |
| FPN_SMH | -0.008 | 0.888 |  | -0.027 | 0.286 | 0.063 |
| RTN_SMH | -0.009 | 0.888 |  | -0.008 | 0.731 | 0.040 |
| SMH_SN | -0.010 | 0.888 |  | 0.021 | 0.394 | 0.082 |
| SMH_SMM | 0.027 | 0.432 |  | 0.012 | 0.628 | 0.134 |
| SMH_VN | 0.002 | 0.895 |  | 0.012 | 0.628 | 0.085 |
| SMH_VAN | 0.045 | 0.175 |  | -0.056 | 0.015 | 0.023 |
| AUN_VAN | 0.035 | 0.098 |  | -0.010 | 0.816 | 0.051 |
| CPN_VAN | -0.057 | 0.012 |  | 0.012 | 0.798 | 0.026 |
| CON_VAN | 0.051 | 0.018 |  | -0.024 | 0.493 | 0.068 |
| DAN_VAN | 0.041 | 0.045 |  | -0.004 | 0.840 | 0.088 |
| DMN_VAN | -0.065 | 0.004 |  | 0.034 | 0.235 | 0.060 |
| FPN_VAN | 0.005 | 0.768 |  | -0.004 | 0.840 | 0.047 |
| RTN_VAN | -0.029 | 0.145 |  | 0.034 | 0.235 | 0.062 |
| SN_VAN | -0.010 | 0.621 |  | -0.016 | 0.798 | 0.085 |
| SMH_VAN | 0.045 | 0.044 |  | -0.056 | 0.031 | 0.023 |
| SMM_VAN | 0.034 | 0.099 |  | -0.007 | 0.840 | 0.037 |
| VAN_VN | 0.017 | 0.405 |  | -0.012 | 0.798 | 0.102 |
| AUN_DAN | -0.010 | 0.628 |  | 0.012 | 0.634 | 0.038 |
| CPN_DAN | 0.035 | 0.127 |  | -0.012 | 0.634 | 0.026 |
| CON_DAN | -0.048 | 0.051 |  | 0.045 | 0.134 | 0.028 |
| DAN_FPN | -0.020 | 0.337 |  | 0.002 | 0.891 | 0.089 |
| DAN_RTN | 0.023 | 0.337 |  | -0.031 | 0.344 | 0.043 |
| DAN_SN | -0.028 | 0.245 |  | 0.042 | 0.134 | 0.031 |
| DAN_SMH | 0.005 | 0.799 |  | 0.022 | 0.634 | 0.028 |
| DAN_SMM | 0.020 | 0.337 |  | -0.015 | 0.634 | 0.077 |
| DAN_VN | -0.036 | 0.122 |  | 0.014 | 0.634 | 0.063 |
| DAN_VAN | 0.041 | 0.075 |  | -0.004 | 0.891 | 0.088 |
| DMN_DAN | 0.048 | 0.043 |  | -0.016 | 0.634 | 0.173 |
| AUN_CPN | -0.021 | 0.350 |  | 0.037 | 0.238 | 0.057 |
| CPN_DAN | 0.035 | 0.124 |  | -0.012 | 0.730 | 0.026 |
| CPN_DMN | -0.034 | 0.124 |  | -0.012 | 0.730 | 0.021 |
| CPN_FPN | -0.009 | 0.698 |  | 0.005 | 0.792 | 0.015 |
| CPN_RTN | 0.041 | 0.086 |  | -0.020 | 0.730 | 0.027 |
| CPN_SN | 0.041 | 0.086 |  | -0.005 | 0.792 | 0.039 |
| CPN_SMH | -0.032 | 0.131 |  | 0.059 | 0.015 | 0.053 |
| CPN_SMM | -0.003 | 0.862 |  | 0.012 | 0.730 | 0.040 |
| CPN_VN | 0.040 | 0.086 |  | -0.007 | 0.792 | 0.016 |
| CPN_VAN | -0.057 | 0.024 |  | 0.012 | 0.730 | 0.026 |
| CON_CPN | 0.019 | 0.378 |  | 0.017 | 0.730 | 0.041 |
| AUN_CON | -0.016 | 0.707 |  | 0.017 | 0.556 | 0.046 |
| CON_CPN | 0.019 | 0.681 |  | 0.017 | 0.556 | 0.041 |
| CON_DAN | -0.048 | 0.051 |  | 0.045 | 0.084 | 0.028 |
| CON_DMN | 0.034 | 0.183 |  | -0.016 | 0.556 | 0.144 |
| CON_FPN | -0.002 | 0.926 |  | -0.003 | 0.875 | 0.044 |
| CON_RTN | 0.003 | 0.926 |  | 0.015 | 0.556 | 0.076 |
| CON_SN | 0.006 | 0.926 |  | 0.019 | 0.556 | 0.032 |
| CON_SMH | -0.020 | 0.681 |  | 0.053 | 0.044 | 0.058 |
| CON_SMM | -0.005 | 0.926 |  | 0.014 | 0.556 | 0.027 |
| CON_VN | -0.009 | 0.926 |  | -0.003 | 0.875 | 0.051 |
| CON_VAN | 0.051 | 0.051 |  | -0.024 | 0.556 | 0.068 |

Adjusted for age, sex, race/ethnicity, site, BMI, marital status, income, parental highest education, handedness and mean motion.

CON= cingulo-opercular network; CPN=cingulo-parietal network; DMN=default mode network

DAN=dorsal attention network; FPN=frontoparietal network; RTN=retrosplenial-temporal network

SN=salience network; VAN=ventral attention network; AUN=auditory network;

SMH=sensorimotor hand network; SMM=sensorimotor mouth network; VN=visual network
